# Mitochondrial Dynamics in Cancer Therapy Response: Context-Dependent Adaptation, Cell Death, and Therapeutic Opportunities

**DOI:** 10.3390/ijms27219538

**Published:** 2026-10-25

**Authors:** Yen Thi Do, Kha Cong Le Bui, Jin Young Kim, So-Jin Shin, Ji Hae Seo

**Affiliations:** 1 Department of Biochemistry, School of Medicine, Keimyung University, Daegu 42601, Republic of Korea; yen94d@gmail.com; 2 Department of Urology B, Hue Central Hospital, Hue 49135, Vietnam; drkhabui.urologist@gmail.com; 3 Department of Internal Medicine, School of Medicine, Keimyung University, Daegu 42601, Republic of Korea; takgu@dsmc.or.kr; 4 Department of Gynecology and Obstetrics, School of Medicine, Keimyung University, Daegu 42601, Republic of Korea

**Keywords:** mitochondrial dynamics, fission and fusion, drug resistance, cancers

## Abstract

Mitochondrial plasticity has emerged as a central determinant of cancer therapy response, enabling tumor cells to adapt to diverse metabolic, environmental, and therapeutic stresses. Among its key features, mitochondrial dynamics contributes to the balance between mitochondrial stress burden and compensatory capacity that shapes cell fate. We propose an adaptive-lethal threshold as a context-dependent conceptual framework describing the transition from reversible mitochondrial dynamics and cellular adaptation to persistent mitochondrial dysfunction and irreversible cell death commitment. Rather than representing a fixed molecular cutoff, this boundary reflects a context-dependent transition in which overall mitochondrial stress can no longer be adequately compensated to maintain cellular homeostasis. Furthermore, how this threshold is defined and reprogrammed in therapy resistance remains unresolved. In this review, we synthesize current advances to propose a framework in which mitochondrial dynamics govern cell fate through threshold-dependent mechanisms. We highlight key pathways across multiple cancer types, including reactive oxygen species (ROS) signaling, mitophagy, metabolic reprogramming, retrograde signaling, evasion of cell death, and interactions with the tumor microenvironment. Importantly, we discuss how context-dependent alterations in these processes can shift the balance between cytoprotective adaptation and irreversible mitochondrial dysfunction, contributing to therapy resistance or cell death. We further highlight emerging therapeutic strategies targeting mitochondrial dynamics and consider how modulation of mitochondrial dynamics can alter mitochondrial stress responses and influence the adaptive-lethal threshold, leading to cell sensitivity. Finally, we discuss key limitations and future directions in defining the context-dependent determinants of the adaptive-lethal transition and their implications for mitochondria-targeted therapies in cancer treatment.

## 1. Introduction

Therapeutic resistance remains a major barrier to effective cancer treatment, reflecting the ability of tumor cells to survive and adapt to diverse therapeutic pressures [1]. Although genetic alterations contribute to drug resistance, cellular plasticity and metabolic adaptation can also enable cancer cells to tolerate treatment and subsequently acquire persistent resistance. Conversely, when adaptive responses fail to maintain cellular homeostasis, therapeutic stress can lead to irreversible dysfunction and cell death [2,3]. Understanding the mechanisms that determine these differential responses is therefore critical for improving therapeutic responses.

Mitochondria have emerged as central regulators of these context-dependent responses because mitochondria integrate metabolic, redox, and cell death signaling. Beyond their classical role in ATP production, mitochondria also orchestrate multiple cellular processes, including metabolic plasticity and redox signaling. By dynamically adjusting mitochondrial metabolism and reactive oxygen species (ROS) levels, cancer cells can sustain proliferation and tolerate stress, contributing to drug resistance. However, excessive mitochondrial dysfunction can instead promote cytotoxic cell death and therapeutic sensitivity [4,5]. Thus, mitochondrial plasticity may influence whether cancer cells adapt to therapy or progress toward irreversible damage.

Among the mechanisms governing mitochondrial adaptation, mitochondrial dynamics—particularly the coordinated processes of fission and fusion—are increasingly recognized as a key determinant of cancer cell response to therapy. Beyond shaping mitochondrial morphology, these processes regulate metabolic reprogramming, mitochondrial quality control, and stress tolerance, whereas dysregulated or excessive mitochondrial dynamics can promote mitochondrial dysfunction, oxidative stress, and cell death [6,7]. Consequently, the impact of mitochondrial dynamics on cancer cell survival and drug response varies substantially across cellular and therapeutic contexts.

This context dependence raises a fundamental question: When does mitochondrial dynamics support adaptation and therapeutic resistance, and when does it contribute to therapeutic cell death? To address this question, we propose an adaptive-lethal threshold as a conceptual framework describing the functional transition between reversible mitochondrial adaptation and persistent mitochondrial dysfunction and cell death commitment. In particular, the threshold is a context-dependent functional transition shaped by the balance between mitochondrial stress and compensatory capacity. In this review, we synthesize current studies to understand how mitochondrial dynamics orchestrates cancer drug resistance through adaptive-lethal-threshold-dependent mechanisms. We focus on key pathways linking mitochondrial dynamics to metabolic plasticity, ROS signaling, mitochondrial quality control, and cell death regulation. Finally, we highlight emerging therapeutic strategies targeting mitochondrial dynamics and altering this balance to overcome resistance in cancer treatment.

## 2. Mitochondrial Dynamics and the Adaptive-Lethal Threshold to Therapy

### 2.1. Mitochondrial Fission−Fusion Homeostasis

**Figure 1:**
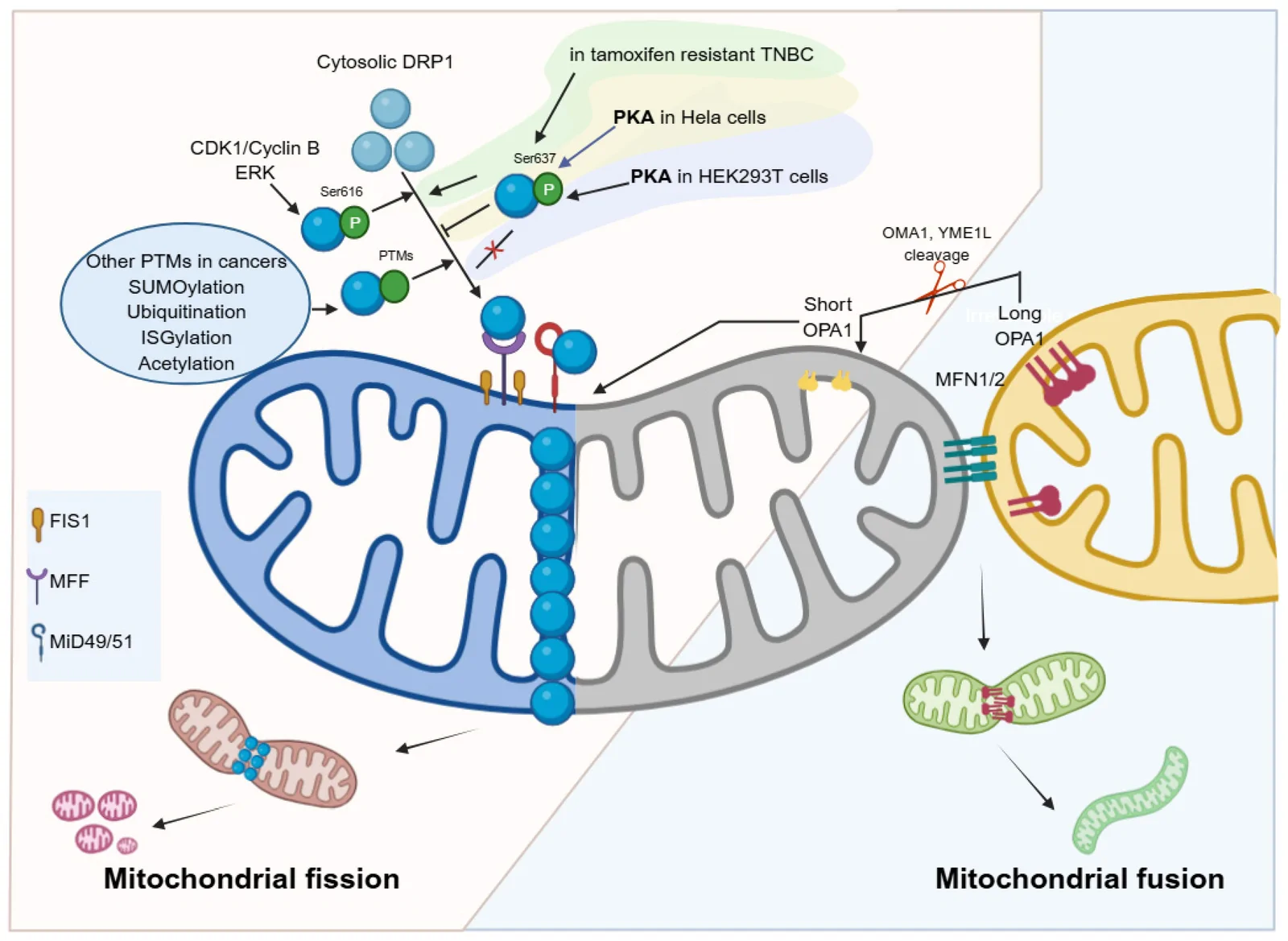
Mitochondrial remodeling is tightly regulated by dedicated molecular machinery. Mitochondrial fission is driven primarily by the translocation of DRP1 from the cytosol to the outer mitochondrial membrane (OMM). DRP1 is recruited by adaptor proteins FIS1, MFF, and MiD49/51, where it oligomerizes to form a contractile ring. This process is tightly regulated by PTMs: phosphorylation at Ser616 (via CDK1/Cyclin B, ERK) or at Ser637 in tamoxifen-resistant TNBC. However, PKA-dependent phosphorylation at Ser637 inhibits mitochondrial fission in Hela cells, but alone does not affect mitochondrial dynamics in HEK293T cells. Other modifications, including SUMOylation, Ubiquitination, ISGylation, and Acetylation may further modulate DRP1 recruitment and stability in cancer. Mitochondrial fusion is a two-step process involving the merging of the outer and inner membranes. MFN1/2 mediate OMM fusion by forming tethering complexes between adjacent mitochondria. OPA1 regulates inner mitochondrial membrane (IMM) fusion. OPA1 activity depends on the balance between long (L-OPA1) and short (S-OPA1) isoforms, which is regulated by the proteases OMA1 and YME1L. While an appropriate L-OPA1/S-OPA1 balance supports mitochondrial fusion, excessive cleavage leads to mitochondrial fragmentation.

Mitochondrial dynamics is governed by the coordinated cycles of fission and fusion, which determine a highly interconnected mitochondrial network, distribution, and function. Under physiological conditions, a dynamic equilibrium between these opposing processes maintains efficient energy production, the exchange of metabolites and mitochondrial DNA (mtDNA), and mitochondrial quality control through the removal of damaged mitochondrial components [8]. This dynamic remodeling is tightly regulated by dedicated molecular machinery (Figure 1). Mitochondrial fission, primarily driven by DRP1 and its adaptors, including FIS1, MFF, MiD49/51, facilitates the segregation of dysfunctional mitochondria and enables their clearance through mitophagy. In contrast, mitochondrial fusion, mediated by MFN1/2 and OPA1, promotes the mixing of mitochondrial contents, thereby diluting damaged components and supporting metabolic efficiency [6,8]. Importantly, fusion and fission are not merely structural processes but are tightly coupled to mitochondrial function, allowing cells to rapidly adjust mitochondrial architecture in response to environmental and intracellular cues.

#### 2.1.1. Dynamin-Related Protein (DRP1)

Mitochondrial fission is primarily orchestrated by DRP1, a cytosolic GTPase that translocates to the outer mitochondrial membrane to drive membrane constriction and mitochondrial division. Upon activation, DRP1 is recruited to mitochondria through adaptor proteins such as FIS1, MFF, and MiD49/51, where it oligomerizes into ring-like structures and then constricts the mitochondrial membrane in a GTP-dependent manner. The activity of DRP1 is tightly regulated by multiple post-translational modifications (PTMs), allowing cells to dynamically adjust mitochondrial fission in response to metabolic and stress signals [9].

Among these modifications, phosphorylation is the most extensively studied mechanism. Phosphorylation of DRP1 at Ser616 by kinases such as CDK1/cyclin B [10] or ERK [11] is generally associated with increased DRP1 activity and mitochondrial fission. In contrast, the functional consequence of Ser637 phosphorylation is highly context-dependent. PKA-dependent phosphorylation at Ser637 has been reported to inhibit DRP1 GTPase activity and suppress mitochondrial fission in cervical cancer cells [12]. In HEK293T cells, however, PKA-dependent phosphorylation at Ser637 alone does not determine DRP1 mitochondrial recruitment. Both phosphomimetic and phosphor-deficient DRP1 variants retained mitochondrial localization, whereas MFF and MiD49/51 contributed to DRP1 recruitment [13]. Conversely, phosphorylation at Ser637 has also been reported to be associated with mitochondrial fission in tamoxifen-resistant TNBC [14]. Collectively, these findings indicate that the functional consequences of DRP1 phosphorylation at Ser637 are cellular context-dependent and that this modification alone may be insufficient to predict mitochondrial dynamics. Therefore, the effects of Ser637 phosphorylation should be interpreted in conjunction with DRP1 activity, mitochondrial recruitment, mitochondrial morphology, and additional regulatory factors such as MFF and MiD49/51.

Beyond phosphorylation, DRP1 is also regulated by additional PTMs. SUMOylation has been implicated in regulating DRP1 stability and mitochondrial recruitment [15], whereas ubiquitination by the E3 ubiquitin ligase MARCH5 can influence DRP1 activity and fission [16]. ISGylation at Lys532 [17] or acetylation of DRP1 [18] has also been reported to promote DRP1 mitochondrial recruitment and fission. While S-nitrosylation [19], a redox-dependent modification, and O-GlcNAcylation [20], a metabolic modification, have been reported to modulate DRP1 localization, stability, or activity in neuronal cells, their functional roles in cancer remain to be elucidated.

#### 2.1.2. Mitochondrial Fission Protein 1 (FIS1)

FIS1 is an outer mitochondrial membrane protein that contains a cytosolic tetratricopeptide repeat (TPR)-like domain involved in protein–protein interactions and a C-terminal transmembrane domain that anchors it to mitochondria [21]. Although early studies proposed FIS1 as the primary receptor for DRP1 recruitment, later evidence suggests that its function is more complex and may involve coordination with other adaptors such as MFF and MiD49/51 [22]. However, more recent evidence suggests a prominent role for FIS1 in mitophagy and apoptosis [23,24].

#### 2.1.3. Mitochondrial Fission Factor (MFF)

MFF is a key adaptor protein located on the outer mitochondrial membrane. Unlike FIS1, MFF is widely considered one of the primary receptors that directly mediate DRP1 docking on the mitochondrial surface. Structurally, MFF is anchored to the outer mitochondrial membrane through a C-terminal transmembrane domain, while its cytosolic N-terminal region interacts with DRP1 and facilitates the assembly of DRP1 oligomeric complexes required for membrane constriction [25]. Phosphorylation of MFF at specific serine residues by AMP-activated protein kinase (AMPK) enhances its ability to recruit DRP1 and promote mitochondrial fission [26]. In addition, SUMOylation [27] and other regulatory modifications including succinylation [28], and ubiquitination [29] may influence MFF stability, localization, and its interaction with components of the fission machinery. The peptide inhibitor P259, which specifically disrupts the interaction between DRP1 and MFF, has enabled the investigation of context-dependent roles of DRP1−MFF signaling and its potential as a target for drug development [30].

#### 2.1.4. Mitochondrial Dynamics 49/51 (MiD49/51)

MiD49/51 are outer mitochondrial membrane adaptors anchored to the outer mitochondrial membrane via a C-terminal transmembrane domain, while their cytosolic domains interact with DRP1 and facilitate its assembly at prospective mitochondrial division sites [31]. Unlike other adaptors, MiD49/51 can recruit DRP1 to mitochondria even in the absence of immediate fission, suggesting that they act as regulatory scaffolds that control the spatial organization and activation state of DRP1 oligomers [32]. MiD51 requires ADP as a cofactor bound through a nucleotidyltransferase-like fold binding [33], suggesting a potential link between metabolic status and mitochondrial fission. Interestingly, MiD51 overexpression causes mitochondrial elongation rather than fission in many cells, likely due to the sequestration of DRP1 into inactive oligomeric complexes [34]. Together, these observations indicate that MiD49/51 function not only as DRP1 receptors but also as regulatory scaffolds that fine-tune mitochondrial fission.

#### 2.1.5. Optic Atrophy 1 (OPA1)

OPA1 is a dynamin-related GTPase localized to the inner mitochondrial membrane and serves as a central regulator of mitochondrial fusion. A key regulatory mechanism is the proteolytic processing of OPA1 into long and short isoforms mediated by the inner mitochondrial membrane proteases OMA1 and YME1L. Under physiological conditions, balanced processing maintains an optimal ratio of long and short OPA1 forms required for efficient mitochondrial fusion [35,36]. However, mitochondrial stress can activate OMA1-dependent cleavage, leading to the loss of fusion-competent OPA1 and promoting mitochondrial fragmentation [37]. In addition, loss of OPA1 or excessive OPA1 proteolytic cleavage can perturb cristae junction integrity and facilitate the release of cytochrome *c* during apoptosis [38,39].

#### 2.1.6. Mitofusin-1 and Mitofusin-2 (MFN1/2)

MFN1/2 are large dynamin-related GTPases embedded in the outer mitochondrial membrane that play a central role in mediating mitochondrial fusion. Both proteins contain an N-terminal GTPase domain, two heptad repeat regions (HR1 and HR2), and a transmembrane domain that anchors them to mitochondria. During mitochondrial fusion, MFN1 and MFN2 form homo- or hetero-oligomeric complexes across adjacent mitochondria through interactions between their HR2 domains, thereby tethering and merging the outer mitochondrial membranes [40,41]. Although MFN1 and MFN2 share similar structural features, they exhibit partially distinct functions. MFN1 generally displays stronger GTPase activity and promotes mitochondrial fusion more efficiently [42], whereas MFN2 also regulates mitochondria–endoplasmic reticulum (ER) contact sites that coordinate calcium signaling and lipid exchange between the two organelles [43]. In addition, E3 ubiquitin ligases such as Parkin [44] and MARCH5 [45] can ubiquitinate MFN proteins, promoting their degradation and thereby suppressing mitochondrial fusion during stress and facilitating the removal of damaged mitochondria during mitophagy. Phosphorylation also influences MFN2 function by modulating its protein–protein interactions and its role in mitochondrial–ER tethering [46,47].

### 2.2. Adaptive Mitochondrial Remodeling and Therapeutic Tolerance

In many therapy-resistant cancers, mitochondrial dynamics are selectively reprogrammed to maintain mitochondrial fitness during treatment. Alterations in mitochondrial morphology can optimize mitochondrial bioenergetics and sustain ATP production under metabolic stress. By preserving mitochondrial membrane potential and respiratory capacity, this adaptive remodeling allows tumor cells to maintain energy supply despite chemotherapy-induced damage. For instance, KLF4-induced mitochondrial fusion increases oxidative phosphorylation (OXPHOS) and protects human glioblastoma cells from stress [48].

Mitochondrial remodeling also contributes to the maintenance of mitochondrial quality control and redox equilibrium. For example, in HCC cells exposed to hypoxia, enhanced DRP1-mediated mitochondrial fission promotes mitophagy, which facilitates the selective removal of damaged mitochondria, thereby supporting tumor cell survival under ischemic hypoxic stress [49]. In parallel, adaptive remodeling may be accompanied by elevated antioxidant capacity, allowing cancer cells to maintain ROS within a range compatible with survival [50].

Furthermore, mitochondrial dynamics can also influence the cellular susceptibility to regulated cell death through the induction of mitochondrial outer membrane permeabilization (MOMP), the release of cytochrome *c*, and the activation of caspase-dependent apoptosis. In cervical cancer and osteosarcoma cells, mitochondrial fission promotes minority MOMP by impairing the pro-apoptotic BAX relocalization from mitochondria to the cytosol. This, in turn, increases non-cytotoxic caspase activation and caspase-dependent DNA damage that may promote genome instability and tumorigenesis [51]. These findings suggest that mitochondrial dynamics may also shape cellular responses to therapeutic stress by modulating the balance between stress adaptation and apoptotic signaling, potentially contributing to cellular plasticity.

Collectively, these observations indicate that mitochondrial dynamics can sustain cancer cell survival, thereby promoting therapeutic tolerance and, in some contexts, contributing to drug resistance. However, when adaptive capacity becomes insufficient to maintain mitochondrial homeostasis, persistent mitochondrial dysfunction and therapy-induced cell death may occur.

### 2.3. Mitochondrial Dysfunction and Therapeutic Sensitivity

Paradoxically, when mitochondrial adaptive capacity becomes insufficient, mitochondrial remodeling may no longer preserve homeostasis and instead contribute to cell death commitment. Both excessive fission and persistent fusion can disrupt mitochondrial function and promote cell death in specific cellular contexts. For example, in TNBC cells, treatment with the flavonoid isorhamnetin (IH), combined with chloroquine-mediated inhibition of autophagy, enhanced ROS-mediated DRP1 activation and mitochondrial fission, accompanied by BAX translocation, cytochrome *c* release, and apoptosis [52]. These findings suggest that excessive DRP1-dependent fission, when coupled with impaired mitophagic clearance, can overwhelm mitochondrial quality-control capacity and shift mitochondrial remodeling from an adaptive response toward apoptotic cell death, potentially revealing a therapeutic vulnerability in TNBC. Conversely, in osteosarcoma cells, mitochondrial fission facilitates proper mitochondrial distribution during cell division, whereas persistent mitochondrial hyperfusion disrupts cell-cycle progression. Prolonged mitochondrial fusion can consequently trigger caspase-dependent cell death through activation and cleavage of caspase-8 [53].

Disruption of mitochondrial dynamics balance can initiate multiple forms of regulated cell death. Mitochondrial fission and fusion can modulate susceptibility to apoptosis by regulating MOMP, thereby promoting the release of cytochrome *c* and subsequent caspase activation [54]. Alternatively, severe metabolic and redox imbalance can trigger non-apoptotic cell death programs such as ferroptosis, which is driven by iron-dependent lipid peroxidation [55], or inflammatory cell death pathways including pyroptosis [56]. However, the evidence linking mitochondrial dynamics to pyroptosis in cancer cells remains limited, as most mechanistic studies have been performed in non-cancer models. Overall, the functional consequences of mitochondrial dynamics depend on the extent and duration of remodeling, as well as the metabolic and cellular context.

Collectively, these findings suggest that the outcome of mitochondrial dynamics depends not simply on the extent or direction of fission and fusion, but also on whether mitochondrial homeostasis can be maintained under therapeutic stress. When the burden of mitochondrial stress exceeds the adaptive capacity, persistent mitochondrial dysfunction may increase susceptibility to therapy-induced cell death. This functional transition from adaptive survival to irreversible mitochondrial dysfunction provides the basis for the proposed adaptive-lethal threshold.

### 2.4. The Adaptive-Lethal Threshold: A Conceptual Framework

The context-dependent effects of mitochondrial dynamics on therapeutic response raise an important question: Why can similar changes in mitochondrial dynamics support cell survival in one setting but promote mitochondrial dysfunction and cell death in another? For example, both increased mitochondrial fusion [57] and fission [58] can also preserve mitochondrial components and their function under moderate therapeutic stress. At this stage, increases in mitochondrial reactive oxygen species (mtROS) can remain manageable through NRF2-dependent antioxidant defenses [59] and the glutathione (GSH) system [60]. Conversely, either mitochondrial fission or fusion may increase oxidative stress, thereby triggering therapy-induced cell death [61,62,63]. Thus, the biological consequence of a given mitochondrial dynamic event cannot be determined solely by its direction or magnitude.

To explain this bidirectional behavior, we propose the adaptive-lethal threshold as a functional boundary describing the transition from adaptive mitochondrial dynamics to persistent mitochondrial dysfunction. When mitochondrial stress remains within this adaptive capacity, mitochondrial dynamics can preserve bioenergetic function, redox balance, and mitochondrial quality, thereby compensating for therapy-induced damage and supporting cell survival and therapeutic tolerance. However, when the stress burden increases or the capacity for mitochondrial adaptation decreases, mitochondrial dynamics may be insufficient to maintain homeostasis, increasing susceptibility to cell death. Thus, the adaptive-lethal threshold is a context-dependent functional boundary whose position can be shifted by factors that alter either mitochondrial stress burden or compensatory capacity.

Importantly, the adaptive-lethal threshold does not represent a fixed quantitative cutoff or a specific degree of any individual mitochondrial parameter. Instead, its position is determined by the dynamic relationship between the overall mitochondrial stress burden and the integrated capacity to maintain homeostasis. At the cellular level, the adaptive-lethal transition can therefore be evaluated through the coordinated changes across three main functional domains: mitochondrial function, compensatory capacity, and cell death signaling. Mitochondrial dynamics may influence this transition by regulating these functional domains and thereby affecting whether mitochondrial stress burden remains compatible with cellular adaptation or progresses toward cell death. Previous studies have shown that mitochondrial fusion and fission responses to stress are highly context-dependent and can support either cell survival or cell death [64]. In oral squamous cell carcinoma (OSCC), context-dependent changes in mitochondrial fission further illustrate how similar alterations in mitochondrial dynamics can produce distinct cellular outcomes under different conditions [65]. Based on these considerations, we summarize potential experimental readouts across the different functional domains that may help operationalize the adaptive-lethal transition (Table 1).

This adaptive-lethal framework therefore places mitochondrial dynamics within a broader stress-adaptation continuum. The adaptive-lethal boundary emerges from the dynamic balance between the mitochondrial stress burden and the compensatory capacity of cancer cells, which is shaped by both intrinsic cellular states and extrinsic conditions. Various factors that alter mitochondrial metabolic capacity, redox buffering, or mitochondrial quality control can shift the balance toward either adaptive survival or persistent mitochondrial dysfunction. Understanding these contextual determinants is essential for explaining why similar alterations in mitochondrial dynamics can mediate distinct therapeutic outcomes across cancer types and treatment settings.

### 2.5. Contextual Determinants of the Adaptive-Lethal Transition

The adaptive-lethal boundary emerges from the dynamic balance between the mitochondrial stress burden and compensatory capacity, both of which are shaped by intrinsic and extrinsic determinants. Importantly, these determinants do not independently define a threshold. Rather, they alter either the magnitude of mitochondrial stress or the capacity to compensate for it.

Among the intrinsic determinants, the basal mitochondrial state, oncogenic signaling, tumor suppressors, and the metabolic phenotype can influence the capacity of cancer cells to accommodate therapeutic stress. Genetic alterations in tumor suppressors and oncogenic pathways can alter mitochondrial dynamics and thereby modify the basal capacity of cancer cells to tolerate mitochondrial stress. For example, while the tumor suppressor p53 [76,77] maintains mitochondrial integrity in cancer, loss of p53 disrupts this regulatory balance and enhances ROS-associated signaling, promoting invasive phenotypes [78]. In contrast, oncogenic MYC [79] and KRAS [80] promote mitochondrial fission and increase mtROS; however, induction of mitochondrial fusion improves mitochondrial function and enhances cell survival.

Similarly, metabolic dependence influences the functional consequences of mitochondrial dynamics. For instance, colon cancer cells that rely heavily on OXPHOS and fatty acid oxidation may favor a more fused mitochondrial network that supports efficient energy production and stress resistance during glucose deprivation [66,81,82]. In this context, mitochondrial fusion may enhance bioenergetic fitness and contribute to survival under chemotherapeutic stress. In contrast, highly glycolytic tumors often maintain mitochondrial fission networks yet remain viable by relying less on mitochondrial ATP production and more on aerobic glycolysis [83]. In such settings, increased mitochondrial fission may reflect a rewired metabolic state compatible with survival. Thus, mitochondrial dynamics should be interpreted in the context of intrinsic determinants and therapeutic outcomes.

Metabolic stress as an extrinsic determinant further influences this balance. During the early phases of glucose deprivation, mitochondria undergo transient MFN1/2-driven hyperfusion, which can maintain ATP production and support short-term survival [84]. However, with prolonged nutrient limitation, mitochondrial dynamics may shift toward fission to induce cell death [85]. In the context of amino acid starvation, a similar hyperfusion state is observed to protect mitochondria from autophagosomal degradation and sustain metabolic function [86]. Notably, resupplementation with specific amino acids can further enhance mitochondrial hyperfusion. This response depends on metabolic pathways, including the TCA cycle and purine biosynthesis, indicating that mitochondrial dynamics is closely coupled to the intracellular metabolic state and metabolic flux in response to nutrient availability [87].

Therapeutic exposure represents a major extrinsic determinant of the mitochondrial stress burden and can therefore influence the adaptive-lethal transition. Cisplatin (CP), for example, induces both DNA damage and mtROS production. To adapt to CP-induced stress, some cancer cells rewire mitochondrial dynamics toward fission to enter a resistant state [88,89]. In contrast, another subset of CP-resistant cells exhibits a shift toward fusion to limit ROS accumulation [90,91]. These apparently opposing remodeling responses can both support therapeutic adaptation by modifying mitochondrial dynamics and changing the adaptive-lethal threshold.

**Figure 2:**
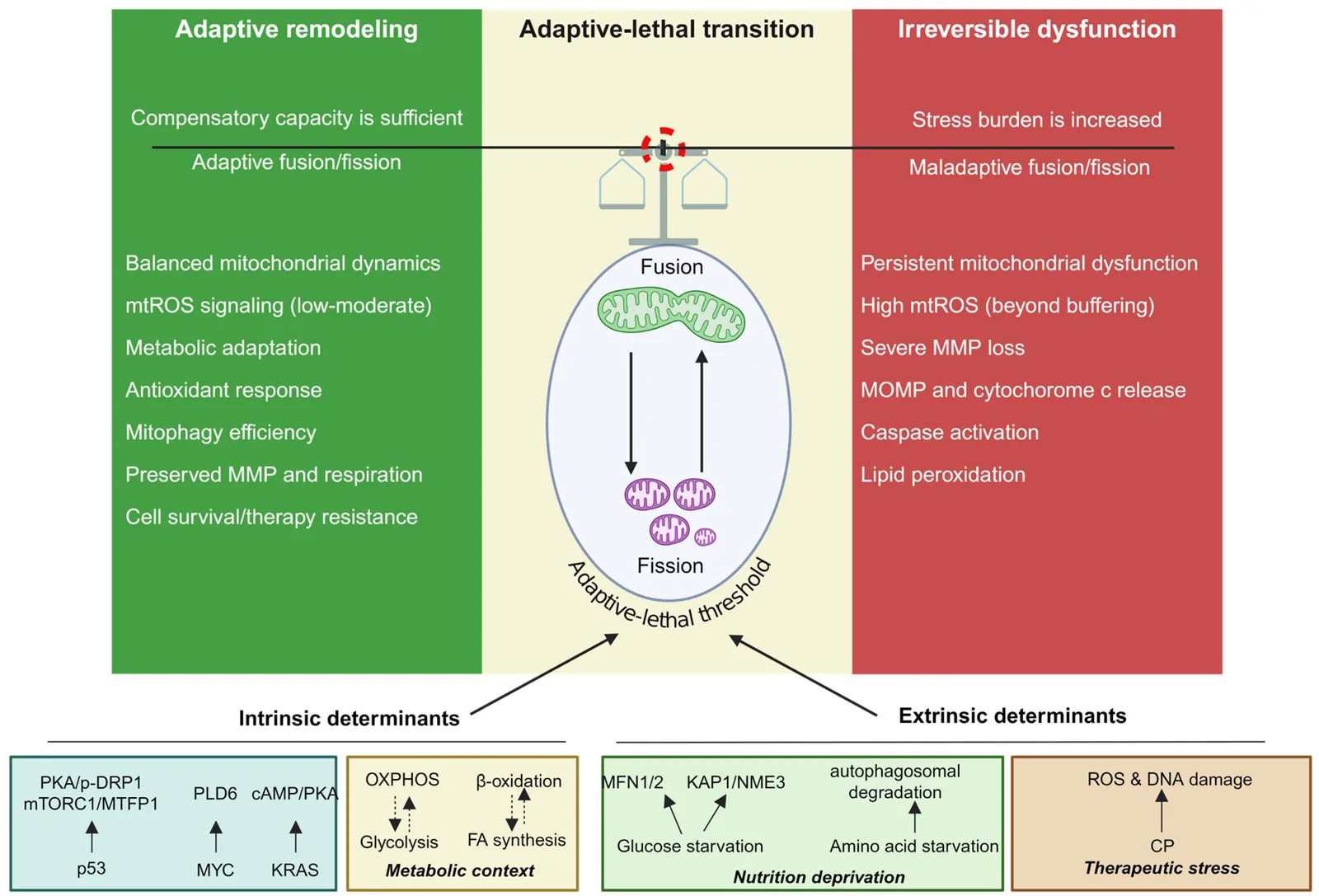
Adaptive-lethal threshold model of mitochondrial dynamics in cancer therapy response. Mitochondrial dynamics function within a context-dependent adaptive-lethal continuum in which both fusion and fission can support either adaptive survival or mitochondrial dysfunction, depending on the balance between mitochondrial stress burden and compensatory capacity. When compensatory capacity is sufficient, adaptive fusion or fission can preserve mitochondrial homeostasis. However, when mitochondrial stress increases or compensatory capacity becomes insufficient, mitochondrial remodeling may become maladaptive, leading to persistent mitochondrial dysfunction. The adaptive-lethal threshold is influenced by intrinsic and extrinsic determinants. Intrinsic determinants include oncogenic signaling (e.g., MYC, KRAS), tumor suppressor pathway (e.g., p53), and metabolic context, whereas extrinsic determinants include nutrient availability and therapeutic agents such as CP. Solid arrows indicate effects supported by previous studies, whereas dashed arrows indicate the potentially opposing effects of metabolic contexts on the adaptive-lethal threshold. The red dashed circle highlights the conceptual adaptive-lethal threshold, representing the transition at which mitochondrial stress exceeds the compensatory capacity of cancer cells.

Importantly, these determinants do not act independently. Rather, they collectively shape the mitochondrial stress burden and compensatory capacity to maintain mitochondrial homeostasis. Consequently, the adaptive-lethal boundary varies across cancer types and is influenced by both intrinsic and extrinsic conditions (Figure 2).

## 3. Mechanistic Networks Linking Mitochondrial Dynamics to Therapy Resistance

### 3.1. ROS-Dependent Modulation

ROS, a heterogeneous group of partially reduced oxygen derivatives, has been extensively studied as a “double-edged sword” in human diseases and cancers, owing to its roles in both promoting and suppressing tumorigenesis. Mitochondria account for most cellular oxygen consumption and are the major source of intracellular ROS. Although ROS are generated by multiple cellular compartments and enzymes, mitochondria have been estimated to contribute approximately 90% of cellular ROS in some studies [92,93]. Importantly, mtROS are both a consequence and a driver of mitochondrial fission, establishing a vicious circuit that reinforces tumor-promoting or tumor-suppressing phenotypes [94].

Mounting evidence positions mitochondrial fission as a central redox integrator during therapeutic stress. Mitochondrial fission forms a self-amplifying loop with elevated ROS, which promotes therapeutic resistance in multiple cancer models. For instance, in hormone-sensitive breast cancer, tamoxifen-resistant cells exemplify this adaptation by increasing mitochondrial fission, thereby leading to elevated ROS leakage due to impaired mitochondrial respiration. The fission-driven ROS leakage is coupled to compensatory antioxidant capacity (SOD2, SIRT3, …) which rewires the redox balance at an adaptive threshold, thereby promoting drug resistance [14]. Similarly, an increased DRP1/MFN1 ratio drives persistent mitochondrial fission, amplifies ROS production, and activates downstream pro-survival signaling pathways including AKT-mediated TP53 degradation and NF-κB activation, ultimately promoting chemoresistance in hepatocellular carcinoma (HCC) [58]. Furthermore, under hypoxic conditions, ROS-induced mitochondrial fission through the dephosphorylation of DRP1 at Ser637 promotes CP resistance in ovarian cancer (OC). Pharmacological inhibition of mitochondrial division reverses this drug resistance [95].

However, the impact of mitochondrial fission on the drug response is highly context-dependent. When excessive fission overwhelms antioxidant defenses, the fission-coupled ROS signaling can shift from adaptive signaling to bioenergetic collapse. These cytotoxic consequences have been well documented in triple-negative breast cancer (TNBC). Enhanced fission augments mtROS accumulation, leading to mitochondrial depolarization and apoptosis, thereby increasing chemosensitivity [96]. Likewise, under hypoxic conditions, ROS-dependent upregulation of MFF via HIF-1α signaling has also been shown to sensitize tumors to CP in a model of head and neck squamous cell carcinoma [97]. Thus, the therapeutic targeting of mitochondrial dynamics requires precise calibration and depends on the tumor subtype and intrinsic antioxidant capacity. A deeper understanding of this redox threshold model may provide a rationale for exploiting mitochondrial dynamics as a strategy to overcome drug resistance.

### 3.2. Mitochondrial Metabolic Plasticity and Adaptation

Cancer cells exhibit profound metabolic plasticity that distinguishes them from normal cells. Increasing evidence indicates that metabolic rewiring is not merely a feature of tumor growth but also a critical adaptive response to therapeutic stress [98]. During treatment, cancer cells frequently remodel metabolic pathways, including glycolysis, lipid metabolism, and amino acid utilization, to mitigate drug-induced oxidative stress, support survival signaling, and promote the persistence of therapy-resistant cell populations such as cancer stem cells [99,100].

Mitochondrial dynamics has emerged as an important upstream regulator of glycolytic flux to support tumor progression and therapeutic resistance. For instance, DRP1 facilitates the progression of castration-resistant prostate cancer (PC) by linking androgen receptor (AR) signaling to mitochondrial metabolic reprogramming. AR signaling upregulates DRP1 expression, promoting the formation of the VDAC-MPC2 complex. This process enhances pyruvate transport into mitochondria, supporting OXPHOS and lipogenesis. Inhibition of DRP1 disrupts this metabolic adaptation and reduces tumor growth, thereby highlighting its potential as a therapeutic target in hormone-resistant PC [101].

Conversely, mitochondrial fusion can also contribute to therapy resistance by reinforcing mitochondrial oxidative metabolism. In gefitinib-resistant lung adenocarcinoma cells, increased expression of the fusion protein OPA1 is associated with mitochondrial fusion and enhanced respiratory activity, indicating a shift toward OXPHOS-dependent metabolism. Suppression of OPA1 reverses these morphological and metabolic adaptations, restoring sensitivity to gefitinib and promoting cytochrome *c*–mediated apoptosis both in vitro and in vivo [102]. Similarly, loss of DRP1 in skeletal muscle induces mitochondrial hyperfusion, leading to impaired fatty acid oxidation and enhanced insulin resistance. Mechanistically, DRP1 deficiency disrupts mitochondrial complex II assembly and activity by limiting the translocation of the assembly factor succinate dehydrogenase assembly factor 2 (Sdhaf2), resulting in succinate accumulation and altered fatty acid β-oxidation [103]. Collectively, these findings suggest that mitochondrial dynamics acts as a metabolic rheostat that enables cancer cells to flexibly switch between glycolytic and oxidative states, as well as between lipid storage and lipid utilization, thereby potentially facilitating drug resistance under therapeutic pressure.

### 3.3. Mitochondrial Retrograde Signaling and Nuclear Reprogramming

Aberrant activation of oncogenic signaling pathways constitutes a major driver of drug resistance in cancer. These pathways transmit signals from mitochondria to the nucleus, orchestrating metabolic programs that enable tumor cells to evade apoptosis, enhance DNA repair, and sustain proliferation under chemotherapeutic stress [6]. Increasing evidence suggests that these signaling cascades are tightly coupled to mitochondrial dynamics. For instance, inhibition of MFN1/2-mediated mitochondrial fusion suppresses the p53-p21 pathway, activating the Ras-Raf-HIF-1α signaling and the expression of glycolytic genes. A metabolic shift from OXPHOS to glycolysis facilitates the acquisition of pluripotency and contributes to the development of drug resistance [5,104]. Similarly, in neural stem cells, disruption of OPA1 or MFN1/2 increases ROS production, which activates NRF2-dependent retrograde signaling. This NRF2-driven transcriptional program suppresses stem cell-like genes while promoting the differentiation of neural stem cells [105].

In a different approach, mitochondrial fusion was enhanced by the intratumoral delivery of membrane-fused mitochondria generated through Pep-1 conjugation (P-Mito), a mitochondrial transplantation strategy. P-Mito enhanced doxorubicin sensitivity and induced cell death involving p53 signaling and AIF nuclear translocation in a TNBC model [106]. These observations suggest that mitochondrial dynamics functions as an upstream regulator of signaling networks, modulating nuclear reprogramming and influencing the therapeutic response. Thus, targeting the mitochondrial–signaling interface may provide a more integrated strategy to overcome drug resistance.

### 3.4. Mitophagy and Mitochondrial Quality Control

Mitophagy, first proposed in 2005, represents a selective autophagic program that orchestrates the removal of damaged or superfluous mitochondria, thereby preserving mitochondrial integrity and maintaining intracellular homeostasis [107]. Mechanistically, mitophagy is governed by either ubiquitin-dependent pathways (most prominently, the PINK1/Parkin-mediated mitophagy) or receptor-mediated mechanisms involving BNIP3, NIX, and FUNDC1, which can be activated under hypoxic or metabolic stress conditions [108]. Importantly, mitochondrial dynamics, particularly DRP1-mediated fission and MFN-driven fusion remodeling, can modulate mitophagic flux [109,110]. This dynamic interplay confers a context-dependent dual role: basal mitophagy may suppress tumor initiation by constraining genomic instability and oxidative damage, whereas stress-induced mitophagy might promote tumor progression and therapy resistance by limiting excessive ROS accumulation and preventing cytochrome *c*–dependent apoptosis during therapeutic challenge [111,112].

Accumulating evidence indicates that chemotherapy-induced stress reprograms mitochondrial dynamics to activate mitophagic flux as an adaptive survival circuit under cytotoxic pressure in diverse cancers. In chemoresistant colorectal cancer, the extracellular release of high-mobility group box 1 protein (HMGB1) activates its receptor for advanced glycation end products (RAGE)–ERK1/2 signaling to phosphorylate DRP1 at Ser616. Enhanced mitochondrial fission triggers autophagy-dependent regrowth of surviving tumor cells. Inhibition of Drp1 phosphorylation by HMGB1 inhibitor and a RAGE blocker significantly restores sensitivity to the chemotherapeutic treatment by suppressing mitophagy [11]. Similarly, oncogenic c-Myc overexpression drives DRP1-mediated mitochondrial fragmentation and p62 aggregation, reinforcing selective mitophagic clearance of damaged mitochondria and suppressing apoptosis in cabozantinib-resistant HCC. Pharmacological inhibition of autophagy or mitochondrial fission in these models restores drug sensitivity, underscoring the pro-survival role of mitophagy during therapeutic stress [113]. Likewise, upregulated mitochondrial fission enhances mitochondrial quality control, potentially allowing cholangiocarcinoma (CCA) cells to adapt to CP-induced stress and evade cell death. Conversely, disruption of mitochondrial balance, by either excessive fission or impaired mitophagy, can resensitize tumors to CP through increased cellular stress and activation of apoptotic pathway [114].

In contrast, CP-resistant OSCC cells display reduced mitochondrial fission and enhanced autophagy. Restoration of ceramide synthase 6 (CS6) expression reestablishes mitochondrial dynamics, impairs mitophagy, and reverses resistance [115]. Collectively, these studies converge on a unifying paradigm in which mitochondrial dynamics are tightly coupled to mitophagy activation, positioning mitochondrial remodeling as a context-dependent determinant of drug response.

However, mitochondrial dynamics can also accompany or result from mitophagic flux. For example, activation of the canonical PINK1/Parkin pathway leads to the rapid ubiquitination and proteasomal degradation of outer membrane fusion proteins (MFN1 and MFN2) [116]. This process can shift mitochondrial morphology toward fragmentation during mitophagy. Furthermore, mitophagic flux can also occur through fission-independent mechanisms, wherein autophagosomes directly engulf elongated organelles, particularly mitochondria, without DRP1 recruitment [117]. Therefore, distinguishing whether mitochondrial structural remodeling acts as a driver or represents a secondary consequence of mitophagic clearance remains essential for interpreting the dynamics–mitophagy relationship.

### 3.5. Regulation of Cell Death Pathways

**Figure 3:**
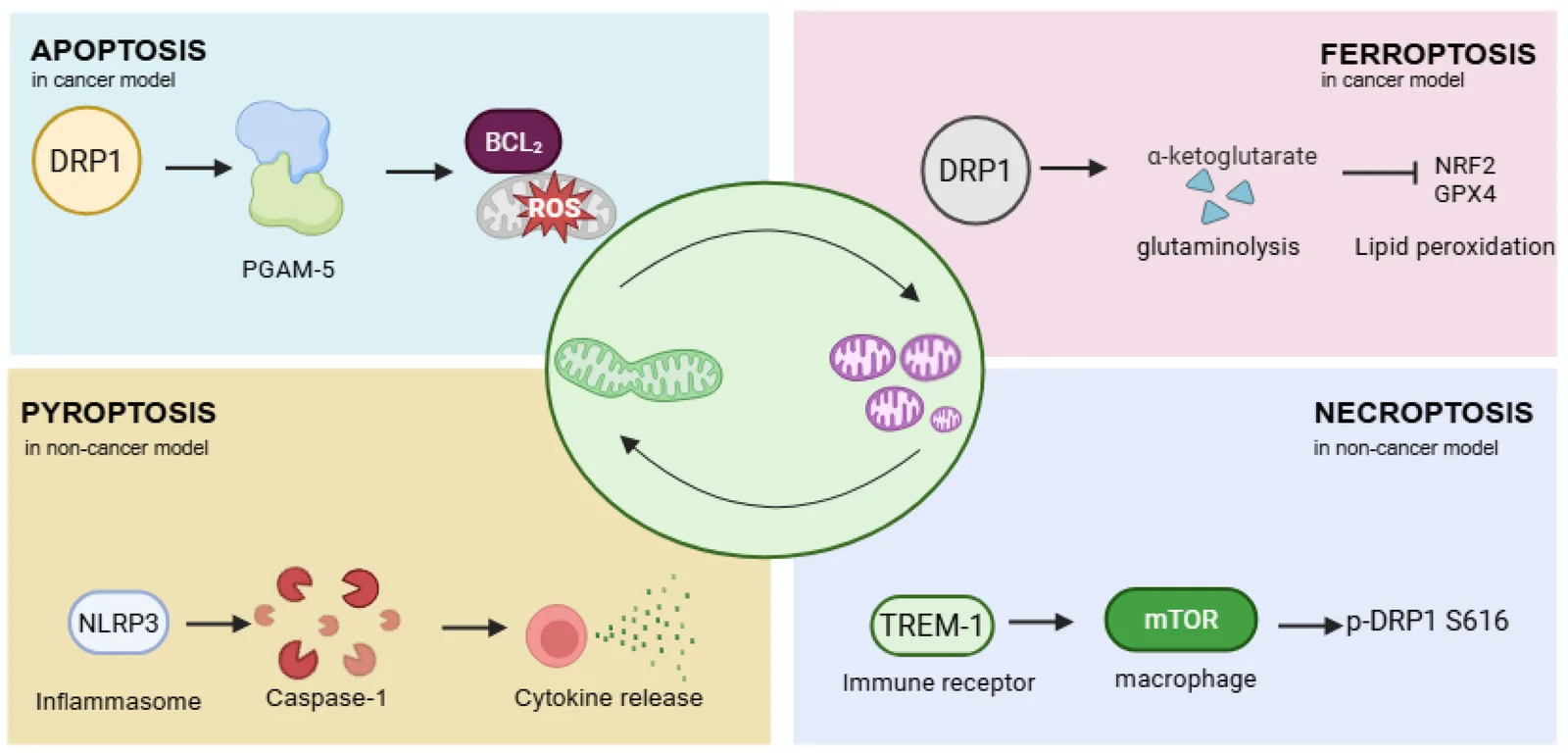
Mitochondrial dynamics coordinate multiple regulated cell death pathways in cancer. Mitochondrial fission and fusion serve as critical hubs for coordinating various RCD pathways in cancer. Apoptosis in cancer model: DRP1 interacts with PGAM5 and BCL-2 to drive mitochondrial permeabilization and ROS accumulation. Ferroptosis in cancer model: DRP1 suppression leads to mitochondrial elongation, which enhances glutaminolysis and α-ketoglutarate levels, overriding NRF2/GPX4 antioxidant defenses. Necroptosis in non-cancer model: The TREM-1/mTOR axis promotes DRP1 Ser616 phosphorylation, triggering excessive fragmentation that amplifies necrotic signaling. Pyroptosis in non-cancer model: Mitochondrial stress and fission facilitate NLRP3 inflammasome assembly and caspase-1 activation, leading to inflammatory cytokine release.

Accumulating evidence indicates that mitochondrial architecture is closely linked to the ability of cancer cells to evade regulated cell death (RCD), a hallmark of therapeutic resistance. Within the tumor microenvironment, multiple forms of RCD—including apoptosis, necroptosis, pyroptosis, and ferroptosis—serve as critical barriers to malignant progression and represent major mechanisms through which anticancer therapies eliminate tumor cells [118]. However, cancer cells frequently develop adaptive strategies that suppress or rewire these death pathways, thereby enabling survival under therapeutic stress [119]. The interplay between mitochondrial dynamics and these four forms of RCD is summarized in Figure 3.

Among these cell death programs, apoptosis remains the most extensively characterized mitochondrial-dependent pathway. Mitochondrial fission, largely mediated by the GTPase DRP1, has long been associated with apoptotic signaling through the promotion of MOMP and the release of cytochrome *c*, leading to caspase activation. DRP1 can interact with BCL-2 family proteins and mitochondrial phosphatases such as PGAM5 to facilitate these processes [54]. However, emerging studies reveal that the relationship between mitochondrial fission and apoptosis is highly context-dependent. In human colon cancer and glioblastoma cells, suppression of DRP1 reduces mitochondrial respiration and inhibits the RHOA/ROCK1 pathway, ultimately increasing apoptotic susceptibility and suppressing tumor cell proliferation [120,121]. These findings highlight that mitochondrial fission not only promotes apoptosis but also functions as an adaptive mechanism that preserves mitochondrial homeostasis and prevents excessive apoptotic signaling in tumor cells.

Ferroptosis, a non-apoptotic form of RCD, is an iron-dependent cell death modality driven by the accumulation of lipid peroxides following glutathione depletion and glutathione peroxidase 4 (GPX4) inactivation. In OSCC stem cells, fragmented mitochondrial networks maintained by DRP1 are required for the preservation of stemness and therapy resistance. Suppression of DRP1 induces mitochondrial elongation, enhances glutaminolysis and α-ketoglutarate production, and ultimately promotes ferroptotic cell death. Conversely, several signaling pathways that regulate oxidative stress and antioxidant responses, such as NRF2-mediated proteasomal degradation of DRP1, can inhibit mitochondrial fission and modulate ferroptosis sensitivity [122].

Mitochondrial dynamics further intersects with inflammatory forms of RCD, including necroptosis and pyroptosis, although direct evidence linking these processes to mitochondrial dynamics in cancer cells remains comparatively limited. Necroptosis is a regulated necrotic pathway that contributes to immune activation and cell death in cancer [123]. Mechanistic evidence from a non-cancer model suggests that mitochondrial fission can act as an upstream modulator of necroptotic signaling. For example, during acute lung injury, activation of the immune receptor TREM-1 promotes necroptosis in macrophages through an mTOR-dependent pathway that enhances DRP1 phosphorylation at Ser616 and drives excessive mitochondrial fragmentation, thereby amplifying necroptotic and inflammatory responses [124]. Similarly, pyroptosis represents another inflammatory form of RCD that has recently gained attention in cancer biology [125]. This process is mediated by inflammasome activation, most commonly through the NLRP3–caspase-1 axis, leading to membrane pore formation and inflammatory cytokine release [56]. The mechanism has been characterized in a non-cancer model and suggests that mitochondrial dynamics acts as a critical upstream regulator of inflammasome activation. In dilated cardiomyopathy, for example, under chemotherapeutic stress, excessive mtROS production can activate DRP1-dependent mitochondrial fission, which facilitates the assembly of the NLRP3 inflammasome and promotes pyroptotic cell death [126]. Although these findings from non-cancer models provide mechanistic insights into how mitochondrial dynamics may intersect with inflammatory death signaling, their relevance to tumor cells and cancer therapy remains to be established.

### 3.6. Tumor Microenvironment (TME)

The TME can be viewed as an integrated bioenergetic ecosystem where mitochondrial dynamics functions in a compartment-specific manner across malignant cells, stromal compartments, and infiltrating immune cells. These distinct cellular compartments impose diverse metabolic and environmental pressures that shape adaptive responses and can influence tumor survival and therapeutic resistance [127].

In malignant tumors, hypoxia represents a major environmental stressor that can reshape mitochondrial dynamics and metabolic adaptation. Under low-oxygen conditions, hypoxia-inducible factor 1α (HIF-1α) activates signaling pathways that promote mitochondrial fission, largely through increased DRP1 activity. This mitochondrial fragmentation facilitates metabolic reprogramming toward glycolysis and enhances the ability of cancer cells to survive oxidative stress and chemotherapy [95,128].

In the stromal niche, cancer-associated fibroblasts (CAFs) can undergo mitochondrial remodeling that supports their metabolic adaptation and tumor-promoting functions. For example, in esophageal squamous cell carcinoma (ESCC), CAFs were reported to exploit mitochondrial fusion through a CD36-MFN2 interaction, which promotes lipid utilization and preserves mitochondrial integrity under metabolic stress [129]. These findings suggest that mitochondrial dynamics in CAFs, rather than only in cancer cells, may contribute to metabolic adaptation within the TME and could represent a potential target for disrupting stromal support of tumor progression.

Crucially, mitochondrial dynamics also shapes the functional state of infiltrating immune cells within the TME. In T cells, the coordination of mitochondrial dynamics supports multiple stages of the antitumor immune response, including activation, differentiation, tumor infiltration, and effector-mediated recognition and elimination of cancer cells. Conversely, dysregulation of mitochondrial dynamics under the metabolically restrictive conditions of the TME can compromise T-cell effector function, thereby contributing to tumor immune evasion [130]. Together, these findings suggest that mitochondrial dynamics may influence therapeutic responses through distinct but potentially interconnected mechanisms within the TME. In cancer cells, mitochondrial remodeling may promote adaptation to hypoxia and treatment-induced stress, whereas mitochondrial changes in CAFs may support stromal metabolic functions. In contrast, dysregulated mitochondrial dynamics in tumor-infiltrating T cells may impair antitumor immune responses. Thus, the therapeutic consequences of targeting mitochondrial dynamics may depend not only on cancer cell-intrinsic responses but also on their effects on stromal and immune compartments. However, direct evidence linking these compartment-specific mitochondrial changes to the coordinated regulation of therapeutic resistance remains limited.

## 4. Therapeutic Implications

Increasing evidence suggests that pharmacological modulation of mitochondrial dynamics may represent a therapeutic strategy for exploiting context-dependent vulnerabilities in cancers. As discussed above, the proposed adaptive-lethal threshold framework suggests that both mitochondrial fission and fusion can support adaptation or contribute to mitochondrial dysfunction and cell death, depending on the cellular context. Therefore, pharmacological inhibition or activation of either process may produce distinct and potentially opposing therapeutic outcomes. This context dependence provides an important rationale for considering the therapeutic targeting of mitochondrial dynamics within the proposed adaptive-lethal threshold framework.

### 4.1. Single Treatment Targeting Mitochondrial Dynamics

Therapeutic strategies targeting mitochondrial dynamics aim to exploit the context-dependent reliance of cancer cells on mitochondrial fission and fusion for survival and adaptation to cellular stress (Table 2). The effects of these interventions depend on the metabolic and stress conditions of cancer cells, as well as their capacity to compensate for alterations in mitochondrial dynamics. Thus, targeting mitochondrial dynamics may either disrupt adaptive survival mechanisms or drive mitochondrial stress beyond the cellular compensatory capacity of cancer cells, consistent with the proposed adaptive-lethal threshold framework.

#### 4.1.1. Targeting Mitochondrial Fission-Dependent Adaptation

Mitochondrial fission can function as an adaptive response that enables cancer cells to maintain metabolic flexibility, preserve mitochondrial quality control, and tolerate persistent cellular stress. In such contexts, inhibiting the fission machinery may impair this compensatory capacity and expose an underlying vulnerability. Accordingly, pharmacological and genetic approaches targeting different components or regulatory mechanisms of the mitochondrial fission machinery have been investigated as potential anticancer strategies.

Among these approaches, DRP1 has received considerable attention because of its central role in mitochondrial constriction and division. Mdivi-1 is one of the most widely used pharmacological tools to interfere with DRP1 activity or its regulatory mechanisms. Mdivi-1 has been reported to reduce mitochondrial fission and suppress cancer cell survival in several tumor models. While Mdivi-1 enhances the sensitivity of OC and CP-resistant OC to death receptor ligands, including TRAIL, FAS ligands, and TNF-alpha [131], Mdivi-1 exerts cytotoxic effects by decreasing oxidative metabolism and triggering death receptor-mediated apoptosis in other hyper-proliferative cancers, including lung and colon cancers [132]. Similarly, Mdivi-1-mediated inhibition of mitochondrial fission has been linked to the modulation of the NF-κB pathway and induction of apoptosis in thyroid cancer [133] and an enhanced TRAIL-induced cell death pathway in melanoma [134]. However, these findings should be interpreted with caution because Mdivi-1 can inhibit mitochondrial complex I activity and alter ROS levels independently of DRP1 [135,136]. Therefore, pharmacological effects observed with Mdivi-1 alone cannot be considered definitive evidence of DRP1 dependence.

Further approaches targeting DRP1 or its regulatory interactions have also been explored. Drpitor1a, which suppresses the GTPase activity of DRP1, reduced mitochondrial fission and promoted apoptosis in NSCLC and TNBC models [137]. Another approach involves honokiol–DCA single treatment, which inhibits DRP1 phosphorylation at Ser616 and triggers apoptosis in Vemurafenib-resistant melanoma [138], and FRAG-i, which directly interacts with the DRP1 GTPase domain but does not alter mitochondrial membrane potential, ROS levels, or cytotoxicity in lung cancer [139]. Dynasore, a non-selective inhibitor of dynamin-family GTPases including DRP1, has likewise been reported to suppress mitochondrial fission and inhibit proliferation in lung cancer cells [140].

Natural compounds have also been investigated as modulators of mitochondrial fission. Hesperetin and naringenin, two natural flavanones, were reported to interact with the GTPase-binding region of DRP1 and suppress its expression and activity, reducing OXPHOS, and inducting ER stress and apoptosis in multiple myeloma models [141]. Collectively, these studies support the possibility that interfering with fission-dependent adaptation can impair tumor proliferation and drug resistance, although the specificity and mechanism of individual pharmacological agents require careful consideration.

#### 4.1.2. Targeting Mitochondrial Fusion-Dependent Adaptation

Conversely, disrupting mitochondrial fusion may represent a therapeutic vulnerability in tumors that rely on a highly fused mitochondrial state to maintain mitochondrial integrity and metabolic fitness. Among the fusion regulators, OPA1 has emerged as a potential therapeutic target. Pharmacological inhibition of OPA1 with MYLS22 disrupts mitochondrial fusion and compromises mitochondrial respiration and cellular fitness in acute myeloid leukemia (AML) models [63]. Another OPA1 GTPase inhibitor, Opitor-0 decreases mitochondrial respiration and increases cell death in metastatic TNBC [142].

Other approaches have targeted mitofusins to interfere with mitochondrial fusion. MFI8, a small molecule that directly binds the HR2 domain of MFN2, inhibits MFN2-mediated mitochondrial fusion and reduces mitochondrial respiration and ATP production, ultimately promoting apoptosis. However, the available evidence for MFI8 is primarily derived from MEFs, and therefore its relevance as an anticancer therapeutic strategy remains to be established [143]. Overall, these studies suggest that mitochondrial fusion can represent a therapeutic vulnerability in selected tumor contexts. Its inhibition may disrupt mitochondrial adaptation and promote cell death when compensatory capacity is limited.

#### 4.1.3. Inducing Mitochondrial Fission and Dysfunction

In contrast to inhibiting mitochondrial fission, mitochondrial fission can also be therapeutically induced when excessive fission compromises mitochondrial function. Oroxylin A provides an example of this context-dependent approach in HCC. Under glucose-restricted conditions, Oroxylin A promotes a fission-dominant mitochondrial state by increasing the expression of the fission protein FIS1 and DRP1 phosphorylation at Ser616, while reducing the levels of the fusion proteins OPA1 and MFN1 and DRP1 phosphorylation at Ser637. These alterations in mitochondrial dynamics were accompanied by inhibition of the SIRT1/PGC-1α/PDK2/PARL axis and induction of apoptosis, with antitumor effects. Thus, Oroxylin A shifts the mitochondrial dynamic balance toward fission and disrupts mitochondrial homeostasis under metabolic stress [144].

Similar effects have been observed with other natural compounds. Baicalein promotes DRP1-associated mitochondrial fission and increases mtROS in tamoxifen-resistant TNBC, whereas silibinin induces DRP1-dependent mitochondrial fission and mitochondrial dysfunction in cervical cancer, ultimately promoting cell death. These findings suggest that pharmacological induction of mitochondrial fission may be effective when sustained fission overwhelms the capacity of tumor cells to maintain mitochondrial homeostasis.

### 4.2. Combination Strategies with Other Therapies

Modulation of mitochondrial dynamics has been investigated as a strategy to enhance the response to conventional, targeted, and immune-based therapies (Table 3). However, the therapeutic consequences of altering mitochondrial dynamics are highly context-dependent and do not consistently favor either inhibition or activation of mitochondrial fission or fusion. This apparent flexibility can be interpreted within the proposed adaptive-lethal threshold framework, whereby the same mitochondrial remodeling may support adaptive survival under one cellular context but exceed the cellular stress-buffering capacity and promote lethal dysfunction under another. Importantly, most available evidence remains derived from in vitro or preclinical in vivo models. Thus, pharmacological targeting of mitochondrial dynamics requires further investigation in a context-dependent manner, with consideration of the cellular stress state and the position of cancer cells relative to the adaptive-lethal threshold.

**Table 2:** Targeting mitochondrial dynamics in cancer: single strategies.

No.	Agents/Gene Intervention	Mode of Action	Genetic Intervention	Mitochondrial Dynamics Effects	Downstream Key Mechanisms	Cancer Model	Evidence/Model Type	Ref
1	Mdivi-1	Pharmacological modulator of fission, originally characterized as DRP1 inhibitor	Knockout DRP1	Mitochondrial fission ↓	Enhance sensitivity to death receptor ligands	OC and CP resistant OC	In vivo model	[131]
			Knockout DRP1		Decrease oxidative metabolism; induce death receptor-mediated apoptosis	Lung and colon cancer	In vitro model	[132]
			Knockdown DRP1		Regulate NF-κB pathway and induce apoptosis	Thyroid cancer	In vitro model	[133]
			Knockdown DRP1		Induce TRAIL-induced cell death pathway	Melanoma	In vitro model	[134]
2	Drpitor1a	Suppress the GTPase activity of DRP1	Knockdown DRP1	Mitochondrial fission ↓	Induce apoptosis	NSCLC, TNBC	In vivo model	[137]
3	Honokiol DCA	Inhibit the phosphorylation of DRP1 at Ser616	Knockdown DRP1	Mitochondrial fission ↓	Increase ROS production and induce apoptosis	Vemurafenib-resistant Melanoma	In vivo model	[138]
4	FRAG-i	Bind directly to the Drp1 GTPase domain and stabilize the protein	-	Mitochondrial fission ↓	Does not impair membrane potential, ATP, ROS	Lung cancer	In vitro model	[139]
5	Dynasore	A small-molecule non-selective inhibitor of the GTPase activity of dynamin 1, dynamin 2, and DRP1	-	Mitochondrial fission ↓	Inhibit proliferation and induce apoptosis	Lung cancer	In vitro model	[140]
6	Hesperetin and Naringenin	Accommodate within the GTPase-binding site of Drp1, inhibiting Drp1 expression and activity	Knockdown DRP1	Mitochondrial fission ↓	Downregulate of master transcriptional activators (SREBF-1, c-MYC) of lipogenesis genes, trigggering ER stress and apoptosis	Myeloma	In vivo model	[141]
7	MYLS22	Pharmacological inhibition of OPA1	MFN2 overexpression	Mitochondrial fusion ↓	Disrupt mitochondrial respiration, ROS production, and cell cycle arrest	Acute myeloid leukemia	In vivo model	[63]
8	Opitor-0	A OPA1 GTPase inhibitor	Knockout OPA1	Mitochondrial fusion ↓	Decrease mitochondrial respiration and increase cell death	TNBC	In vitro model	[142]
9	MFI8	Direct binding to the recombinant HR2 domain of MFN2	Knockout MFN2	Mitochondrial fusion ↓	Reduce respiration and mitochondrial ATP production and induce apoptosis	MEFs	In vitro model	[143]
10	Oroxylin A	Upregulate the pro-fission protein FIS1 and p-DRP1-Ser616 Downregulate the pro-fusion proteins OPA1, MFN1 and p-DRP1-Ser637	Knockout OPA1	Mitochondrial fusion ↓, fission ↑	Inactivate SIRT1/PGC-1α/PDK2/PARL axis and induce apoptosis under glucose restriction condition	HCC	In vivo model	[144]
11	Baicalein	Increase Drp1 and Fis1 expression	-	Mitochondrial fission ↑	Suppress HIF-1α expression, reduce the elevated aerobic glycolysis, and induce ROS-dependent apoptosis	Tamoxifen-resistant TNBC	In vivo model	[145]
12	Silibinin	Increase the expression of Drp1	Knockdown DRP1	Mitochondrial fission ↑	Induce G2/M Cell Cycle Arrest and induce apoptosis	Cervical cancer	In vivo model	[146]

↑: Increase, ↓: Decrease.

**Table 3:** Targeting mitochondrial dynamics in cancer: combination strategies.

No.	Agents/Gene Intervention	Mode of Action	Genetic Intervention	Mitochondrial Dynamics Effects	Downstream Key Mechanisms	Combination Partner	Cancer Model	Evidence/Model Type	Ref
1	Mdivi-1	Pharmacological modulator of fission, originally characterized as DRP1 inhibitor	Knockout DRP1	Mitochondrial fission ↓	Promote mitochondrial dysfunction and swelling; enhance MOMP and mitochondrial apoptotic signaling in a Bax/Bak-independent manner	CP	TNBC, NSCLC, glioblastoma cells	In vitro model	[147]
2			Knockdown DRP1	Mitochondrial fission ↓	Downregulate c-FLIP protein expression and induce caspase-dependent apoptosis	CP	Renal carcinoma	In vitro model	[148]
3			Knockout DRP1	Mitochondrial fission ↓	Enhance sensitivity to death receptor ligands	CP	CP resistant OC	In vivo model	[131]
4			Knockdown DRP1	Mitochondrial fission ↓	Increase ROS production, induce apoptosis- and autophagy-mediated cell death	PTX	PTX-resistant TBNC	In vivo model	[149]
5	Quercetin	Induce DRP1-mediated mitochondrial fission	-	Mitochondrial fission ↑	Regulate autophagic flux, increase ROS production and induce sensitivity of CRC to 5-FU	5-FU	CRC	In vitro model	[150]
6	Mdivi-1	DRP1 inhibition	-	Mitochondrial fission ↓	Revert the paradoxical activation of MAPK/ERK pathway elicited by Vemurafenib and inhibit cell proliferation	Vemurafenib	NRAS-mutant melanoma	In vitro model	[151]
7	Mdivi-1	DRP1 inhibition	-	Mitochondrial fission ↓	Induce apoptosis	Sorafenib	HCC	In vitro model	[152]
8	Mdivi-1	Modulate FIS1-DRP1-dependent mitochondrial fission	Knockdown FIS1	Mitochondrial fission ↓	Inhibit migration and invasion	Crizotinib	HCC	In vivo model	[153]
9	Honokiol DCA	Inhibit the phosphorylation of DRP1 at Ser616	Knockdown DRP1	Mitochondrial fission ↓	Increase ROS production and induce apoptosis	Vemurafenib	Vemurafenib-resistant Melanoma	In vivo model	[138]
10	Baicalein	Increase Drp1 and Fis1 expression	-	Mitochondrial fission ↑	Suppress HIF-1α expression, reduce the elevated aerobic glycolysis, and induce ROS-dependent apoptosis	Tamoxifen	Tamoxifen-resistant TNBC	In vivo model	[145]
11	MYLS22	Pharmacological inhibition of OPA1	-	Mitochondrial fusion ↓	Induce cytochrome *c* release and apoptosis	Gefitinib	gefitinib-resistant lung cancer	In vivo model	[102]
12	Opitor-0	A OPA1 GTPase inhibitor	-	Mitochondrial fusion ↓	Enhance cytochrome *c* release and induce apoptosis	ABT-737	TNBC	In vitro model	[154]
13	Opitor-0	A OPA1 GTPase inhibitor	Knockdown OPA1	Mitochondrial fusion ↓	Increase glutamine dependence Sensitize to ferroptosis	Venetoclax	AML	In vivo model	[155]
14	Mdivi-1	DRP1 inhibition	Knockdown DRP1	Mitochondrial fission ↓	Modulate the PD1-ERK/DRP1 pathway and potentiate T-cell antitumor immune response	Pembrolizumab	Lung cancer	In vivo model	[156]

↑: Increase, ↓: Decrease.

#### 4.2.1. Combination with Chemotherapy

Combination strategies that target mitochondrial dynamics alongside conventional therapies have emerged as a promising approach to overcome drug resistance. In some models, suppression of DRP1-dependent mitochondrial fission has shown synergistic effects when combined with chemotherapeutic agents. Mdivi-1, originally characterized as a DRP1 inhibitor, increased the response to CP in TNBC, NSCLC, glioblastoma, and OC models by promoting mitochondrial dysfunction and enhancing mitochondrial apoptotic signaling [131,147]. Similarly, combined treatment with Mdivi-1 and CP also downregulated c-FLIP expression transcriptionally and induced activation of caspase-dependent cell death in renal carcinoma cells, but not in normal cells [148]. In PTX-resistant TNBC, Mdivi-1 treatment sensitized TNBC by increasing ROS production and activating apoptotic- and autophagy-associated cell death pathways [149]. These findings suggest the possibility that reducing mitochondrial fission may increase the vulnerability of chemotherapy-resistant cancer cells.

However, mitochondrial fission can also be therapeutically exploited in the opposite direction. Quercetin increased DRP1-mediated mitochondrial fission, ROS production, and autophagic flux, thereby enhancing the sensitivity of CRC to 5-FU treatment [150]. Together, these findings indicate that both suppression and induction of mitochondrial fission may enhance chemotherapy sensitivity, supporting the concept that the therapeutic effect of mitochondrial remodeling depends on the cellular context.

#### 4.2.2. Combination with Targeted Therapy

Mitochondrial dynamics has also been investigated in combination with molecularly targeted therapies, particularly in tumors that undergo metabolic adaptation during treatment. In NRAS-mutant melanoma, Mdivi-1 enhanced the antiproliferative effect of Vemurafenib by suppressing mitochondrial fission and reversing paradoxical MAPK/ERK pathway activation [151]. In Vemurafenib-resistant melanoma, honokiol DCA inhibited DRP1 Ser616 phosphorylation and enhanced ROS production and apoptosis when combined with Vemurafenib [138]. Similarly, a combination of Mdivi-1 and sorafenib also induced apoptosis in HCC [152], whereas another combination of Mdivi-1, which was used as a modulator of FIS1-DRP1-dependent mitochondrial fission, with Crozotinib inhibited migration and invasion in HCC model [153]. Although FIS1 has been implicated in the regulation of mitochondrial dynamics, mitophagy, apoptosis, and stress signaling, its role in DRP1 recruitment is context-dependent. Thus, targeting the FIS1-DRP1 axis should not be considered a universal approach to inhibit DRP1 recruitment.

Conversely, induction of mitochondrial fission may also enhance the response to targeted therapy in specific cellular contexts. In tamoxifen-resistant TNBC, baicalein increased DRP1 and FIS1 expression and promoted mitochondrial fission, inducing ROS-dependent apoptosis [145]. These findings further support the context-dependent manner of mitochondrial fission, whereby either suppression or induction of fission may increase therapeutic vulnerability.

Targeting mitochondrial fusion provides another therapeutic strategy in tumors that depend on sustained mitochondrial integrity. Pharmacological inhibition of OPA1 with MYLS22 restored Gefitinib sensitivity in Gefitinib-resistant lung cancer, accompanied by cytochrome *c* release and apoptosis [102]. Similarly, inhibition of OPA1 with Opitor-0 sensitized TNBC cells to the BCL-2 inhibitor, ABT-737 [154]. In AML, Opitor-0 disrupted OPA1-dependent mitochondrial fusion, increased glutamine dependence, and enhanced ferroptotic sensitivity, thereby improving the response to Venetoclax [155]. Together, these findings indicate that both fission and fusion can represent therapeutic vulnerabilities, but the direction of mitochondrial dynamics modulation needs to be considered in relation to the cellular context.

#### 4.2.3. Combination with Immunotherapy

Mitochondrial dynamics may also influence the metabolic fitness and antitumor activity of immune cells, providing a potential rationale for combining mitochondrial-targeting strategies with immunotherapy. For example, increased DRP1-mediated fission in T cells enhanced T-cell activation, cytokine secretion, CD8+ T-cell infiltration, and the antitumor effect of PD-1 blockade in a lung cancer model. Conversely, combination treatment with Mdivi-1 and PD-1 inhibitor (Pembrolizumab) promoted a memory-like T-cell phenotype and enhanced the antitumor effect in a preclinical lung cancer model [156]. These findings suggest that modulation of mitochondrial fission may enhance the functional persistence of immune cells.

## 5. Conclusions and Future Directions

In summary, mitochondrial dynamics has emerged as a central regulator of the cancer therapeutic response, functioning far beyond the maintenance of mitochondrial morphology. As a highly adaptable and interconnected network, mitochondrial remodeling integrates bioenergetic regulation, redox homeostasis, metabolic reprogramming, mitochondrial quality control, retrograde signaling, tumor microenvironmental cues, and multiple forms of regulated cell death. Through these coordinated processes, mitochondrial dynamics enables cancer cells to continuously adapt to therapeutic stress and plays a critical role in the development of treatment resistance. Importantly, the impact of mitochondrial dynamics on cancer cell fate is fundamentally context-dependent. Mitochondrial fusion and fission can both support adaptive or maladaptive responses. Within this framework, we propose the adaptive-lethal threshold as a conceptual representation of the functional boundary between reversible mitochondrial adaptation and persistent mitochondrial dysfunction. When mitochondrial stress remains within the compensatory capacity of the cell, mitochondrial remodeling promotes survival by preserving mitochondrial fitness, maintaining redox equilibrium, supporting metabolic flexibility, and facilitating mitophagy-mediated quality control. However, when mitochondrial stress can no longer be adequately compensated, persistent or maladaptive mitochondrial remodeling, whether involving excessive fission or fusion, can lead to bioenergetic collapse, uncontrolled ROS accumulation, impaired mitochondrial quality control, and susceptibility to context-specific regulated cell death pathways.

Despite growing evidence linking mitochondrial dynamics to cancer therapy response, several fundamental questions remain unresolved. A key challenge is to identify the determinants that shift the balance between adaptive remodeling and persistent mitochondrial dysfunction across different cancer types and therapeutic contexts. Rather than representing a fixed quantitative cutoff, the adaptive-lethal threshold reflects the dynamic relationship between the mitochondrial stress burden and the integrated capacity. Future studies should therefore determine how signaling networks, metabolic states, PTMs, and tumor microenvironmental cues alter this balance over time. Integrating longitudinal measurements of mitochondrial dynamics with functional assessments of respiration, redox status, metabolic adaptation, mitochondrial bioenergetics, and cell fate may help resolve how adaptive responses evolve during treatment and when they become maladaptive. Finally, from a translational perspective, the pharmacological modulation of mitochondrial dynamics represents an emerging therapeutic strategy. Small-molecule inhibitors targeting key regulators such as DRP1 have shown encouraging results in preclinical models, particularly when combined with conventional chemotherapy or targeted therapies. However, as the clinical development of mitochondrial dynamics modulators remains limited, further studies are warranted to evaluate their specificity, toxicity, and therapeutic efficacy.

## 6. Literature Search and Selection

Literature relevant to mitochondrial dynamics, fusion, fission, mitochondrial function, redox signaling, mitophagy, metabolic plasticity, cell death, and therapeutic response in cancer was identified through searches of PubMed and Google Scholar. The literature search covered publications available in the period 2010–2026. Search terms combined relevant keywords, including “mitochondrial dynamics”, “mitochondrial fusion”, “mitochondrial fission”, “mitochondrial ROS”, “mitophagy”, “metabolic reprogramming”, “signaling pathways”, “cell death”, “apoptosis”, “ferroptosis”, “necroptosis”, “pyroptosis”, “tumor microenvironment”, “cancer”, “therapy resistance”, “therapeutic response”, “inhibitor”, “activator”, “oncogene”, “tumor suppressor”. Given the narrative nature of this review, publications were selected based on their relevance to the scope of the review, mechanistic insight, and contribution to the development or support of the concepts discussed. Foundational studies, recent advances, and representative reviews were considered to provide a balanced synthesis of the field.

## Figures and Tables

**Table 1:** Experimental readouts for assessing the adaptive-lethal transition and their interpretive considerations.

No.	Functional Domain	Adaptive State	Irreversible Dysfunction	Interpretive Considerations	Ref
1	Mitochondrial membrane potential (∆ψm)	Preserved/recoverable	Sustained depolarization	Transient ∆ψm loss may reflect reversible stress and does not indicate irreversible cell death by itself.	[66]
2	Respiratory function (OCR)	Maintained OCR/recoverable	Persistent impairment	Strongly depends on substrate availability, cell type, and metabolic state.	[67]
3	mtROS	Controlled/buffered	Persistent excessive ROS	Increased mtROS levels can reflect either adaptive oxidative stress or irreversible mitochondrial damage.	[68,69]
4	Redox buffering (GSH/GSSG)	Preserved antioxidant capacity	Loss of buffering capacity	Reflects cellular redox balance and does not directly establish mtROS levels.	[70]
5	Mitophagic flux	Functional turnover	Impaired or insufficient clearance	Increased flux may indicate either effective quality control or increased mitochondrial damage requiring clearance.	[71]
6	mtDNA release	Limited/controlled	Increased cytosolic mtDNA	Context-dependent and not specific to a single death pathway.	[72]
7	MOMP/cytochrome *c*	Preserved/maintained	Increased MOMP and cytochrome *c* release	Provides strong evidence of apoptotic commitment but does not capture other forms of cell death.	[73]
8	Caspase activation	Minimal	Sustained activation	Provides evidence of apoptotic machinery but does not capture other types of cell death.	[74]
9	Lipid peroxidation	Controlled	Increased	Supports the assessment of ferroptosis but requires context-specific factors.	[75]
10	Clonogenic survival	Recovery after stress removal	Loss of long-term reproductive capacity	Provides functional evidence of reversibility but does not establish a specific mitochondrial mechanism.	[51]

## Data Availability

No new data were created or analyzed in this study. Data sharing is not applicable to this article.

## Abbreviations

The following abbreviations are used in this manuscript:

AMPK	AMP-activated protein kinase
AR	androgen receptor
AIF	apoptosis-inducing factor
CAFs	cancer-associated fibroblasts
CS6	ceramide synthase 6
CCA	cholangiocarcinoma
CP	Cisplatin
DRP1	Dynamin-related protein
ER	endoplasmic reticulum
EMT	epithelial–mesenchymal transition
HCC	hepatocellular carcinoma
HR	heptad repeat regions
HMGB1	high-mobility group box 1
HIF-1α	hypoxia-inducible factor-1α
IMM	inner mitochondrial membrane
mtDNA	mitochondrial DNA
MiD49/51	Mitochondrial dynamics 49/51
MFF	Mitochondrial fission factor
FIS1	Mitochondrial fission protein 1
MFN1/2	Mitofusin-1 and mitofusin-2
NOX	NADPH oxidase
OPA1	Optic atrophy 1
OSCC	oral squamous cell carcinoma
MOMP	outer membrane permeabilization
OMM	outer mitochondrial membrane
OC	ovarian cancer
OXPHOS	oxidative phosphorylation
PTMs	post-translational modifications
PC	prostate cancer
PKA	protein kinase A
ROS	reactive oxygen species
RAGE	receptor for advanced glycation end product
RCD	regulated cell death
Sdhaf2	succinate dehydrogenase assembly factor 2
TPR	tetratricopeptide repeat
TME	Tumor microenvironment
